# Association and Predictive Value Analysis for Resting Heart Rate and Diabetes Mellitus on Cardiovascular Autonomic Neuropathy in General Population

**DOI:** 10.1155/2014/215473

**Published:** 2014-03-18

**Authors:** Zi-Hui Tang, Fangfang Zeng, Zhongtao Li, Linuo Zhou

**Affiliations:** Department of Endocrinology and Metabolism, Huashan Hospital, Fudan University, No. 12 Wulumuqi Mid Road, Building No. 0, Jing'an District, Shanghai 200040, China

## Abstract

*Background.* The purpose of this study was to evaluate the predictive value of DM and resting HR on CAN in a large sample derived from a Chinese population. *Materials and Methods.* We conducted a large-scale, population-based, cross-sectional study to explore the relationships of CAN with DM and resting HR. A total of 387 subjects were diagnosed with CAN in our dataset. The associations of CAN with DM and resting HR were assessed by a multivariate logistic regression (MLR) analysis (using subjects without CAN as a reference group) after controlling for potential confounding factors. The area under the receiver-operating characteristic curve (AUC) was used to evaluate the predictive performance of resting HR and DM. *Results.* A tendency toward increased CAN prevalence with increasing resting HR was reported (*P* for trend <0.001). MLR analysis showed that DM and resting HR were very significantly and independently associated with CAN (*P* < 0.001 for both). Resting HR alone or combined with DM (DM-HR) both strongly predicted CAN (AUC = 0.719, 95% CI 0.690–0.748 for resting HR and AUC = 0.738, 95% CI 0.710–0.766 for DM-HR). *Conclusion.* Our findings signify that resting HR and DM-HR have a high value in predicting CAN in the general population.

## 1. Background

Diabetes mellitus (DM) is a group of metabolic diseases in which a person has high blood sugar, either because the pancreas does not produce enough insulin or because cells do not respond to the insulin that is produced [1]. Globally, as for 2010, an estimated 285 million people had diabetes, with type 2 making up about 90% of the cases [1]. Its incidence is increasing rapidly, and by 2030, this number is estimated to almost double [2]. The prevalence of cardiovascular autonomic neuropathy (CAN) is also rapidly growing in all populations worldwide, particularly in the developing world [3, 4]. The disease is not only a major factor in the cardiovascular complications of DM [5] but also affects many other major segments of the general population, such as the elderly, patients with hypertension (HT), metabolic syndrome (MetS), and connective tissue disorders [3, 6–8]. CAN has become a major health concern in China following recent, rapid changes in lifestyles. In diabetic patients, the prevalence of CAN was 30–60% [5]. Individuals with previously undiagnosed CA dysfunction have an unfavorable cardiovascular risk profile, especially in terms of sudden death, indicating a higher risk of cardiovascular disease [9, 10]. Previous studies indicate that increased resting HR is an early indicator of CAN, and a strong association between HR and CAN was found in diabetic patients [11, 12]. Moreover, resting HR is considered a critical clinical early sign for CAN in diabetic patients. In our previous study, in a Chinese population, DM and resting HR were found to be strongly associated with components of CA function [13].

It is important to clarify the predictive value of DM and resting HR in predicting at the general population level, as this information can help clinicians in the prediction, prevention, and treatment of CAN. However, at the population level, the role of DM or resting HR in predicting CAN has not been well defined. The purpose of this study is to evaluate the predictive value of DM and resting HR for CAN in a large sample derived from a Chinese population.

## 2. Materials and Methods

### 2.1. Study Population

We performed a CAN factor survey on a random sample of the Chinese population. Participants were recruited from rural and urban communities in Shanghai. Survey participants with undiagnosed CAN, aged 30–80 years, were included in this study. A total of 3,012 subjects were invited to a screening visit between 2011 and 2012. Some subjects were excluded from the study to eliminate potential confounding factors that may have influenced their CA function. Briefly, the exclusion criteria were as follows: (1) history or findings of arrhythmia, hyperthyroidism, or hypothyroidism; (2) pregnancy or lactation; and/or (3) serious hepatic or renal dysfunctions (the definition of serious liver or renal disease is that more than 1.5-fold elevation of alanine aminotransferase, aspartate aminotransferase, or direct bilirubin or serum creatinine > 115 *μ*mol/L). Complete baseline data were obtained for 2,092 (69.46%) of the participants. The subjects were interviewed for the documentation of medical histories and medication, history of smoking habits, laboratory assessment of cardiovascular disease risk factors, and standardized examination for CA function. Written consent was obtained from all patients before the study. This study was approved by the Ethics Committee of the Huashan Hospital, Shanghai, China.

### 2.2. Measurement

All study subjects underwent a complete clinical baseline characteristics evaluation after an eight-hour fast, which included: (1) history and physical examination; (2) heart rate and blood pressure; (3) fasting serum glucose and insulin; and (4) fasting plasma lipids. Body mass index (BMI) was calculated with weight in kilograms divided by the square of height in meters. Physicians measured systolic and diastolic blood pressure (BP) values from the left arm while participants were seated. Fasting plasma glucose (FPG) was quantified by the glucose oxidase procedure; HbA1c was measured by ion-exchange high-performance liquid chromatography (HPLC; Bio-Rad, Hercules, CA, USA). The homeostasis model assessment insulin resistance estimate (HOMA-IR) was calculated as serum glucose (mmol/L) multiplied by plasma insulin (U/mL) and divided by 22.5. Serum total cholesterol (TC), high-density lipoprotein (HDL) cholesterol, triglyceride (TG) levels, serum creatinine (SCr), and uric acid (UA) were measured by an enzymatic method with a chemical analyzer (Hitachi 7600-020, Tokyo, Japan). Low-density lipoprotein (LDL) cholesterol levels were calculated using the Friedewald formula. The day-to-day and interassay coefficients of variation at the central laboratory in our hospital for all analyses were between 1% and 3%.

Short-term HRV has good reproducibility and is more practical in its application. In our large-scale population-based study, this test was used to evaluate CA function. HRV was measured noninvasively by power spectral analysis. Before CA function assessment, participants must avoid alcohol, smoking, and coffee, tea, or other sources of caffeine for 24 hours so as not to influence their resting status. Subjects were studied while awake in the supine position after 20 minutes of rest. Testing times were from 8:00 to 11:00 a.m. A type-I FDP-1 HRV noninvasive detecting system was used with software version 2.0 (Department of Biomedical Engineering of the Fudan University, Shanghai, China). Electrocardiography, respiratory signals, and beat-to-beat blood pressure were continually and simultaneously recorded for 15 minutes through an electrosphygmograph transducer (HMX-3C placed on the radial artery of the dominant arm) and an instrument respiration sensor. Short-term HRV analysis was performed for all subjects using a computer-aided examination and evaluation system for spectral analysis to investigate changes in autonomic regulation. The reproducibility and day-to-day coefficients of variation for above methods were less than 5%.

### 2.3. Definition

HT was defined as BP ≥ 140/90 mmHg or a history of hypertension medication. BMI was classified based on the Chinese criteria: normal as BMI < 24.0 kg/m^2^; overweight as 24.0 kg/m^2^ ≤ BMI < 28.0 kg/m^2^; and obese as BMI ≥ 28.0 kg/m^2^. High FPG was defined as FPG ≥ 5.6 mmol/L. Center obesity was defined using ethnicity-specific values for waist circumference (WC) of ≥90 cm in men and ≥80 cm in women [14]. TG was defined as TG ≥ 1.7 mmol/L. HDL was defined as HDL < 0.9 mmol/L in men and HDL < 1.0 mmol/L in women. MetS was diagnosed according to the updated National Cholesterol Education Program/Adult Treatment Panel III criteria (WHO Western Pacific Region obesity criteria) in individuals meeting three or more of the following [14]. DM was defined by oral glucose tolerance test (OGTT) and either HbAlc ≥ 6.5% or the use of insulin or hypoglycemic medications. CAN was diagnosed based on at least two abnormal cardiovascular autonomic reflex test results [5]. For analysis, the main independent variables of resting HR were categorized into four groups (codes 1 for <65; 2 for 65–75; 3 for 76–85; and 4 for >85 bpm) and DM-HR (a categorized variable with combined information of DM and resting HR) was categorized into eight groups (codes 1 for HR <65 bpm and non-DM; 2 for HR <65 bpm and DM; 3 for HR 65–75 bpm and non-DM; 4 for HR 65–75 bpm and DM; 5 for HR 76–85 bpm and non-DM; 6 for HR 76–85 bpm and DM; 7 for HR > 85 bpm and non- DM; and 8 for HR > 85 bpm and DM).

### 2.4. Statistical Analysis

The Kolmogorov-Smirnov test was used to determine whether continuous variables followed a normal distribution. Variables that were not normally distributed were log-transformed to approximate normal distribution for analysis. The results are expressed as the mean ± SD or median, unless otherwise stated. The characteristics of the subjects according to CAN groups were assessed using one-way analysis of variance (ANOVA) for continuous variables and the *χ* ^2^ test for categorical variables. Univariate linear regression was performed to determine the variables associated with CAN and to estimate confounding factors possibly disturbing the relationship between CAN and DM or HR. Multivariate logistic linear regression (MLR) was carried out to determine the independent contributions of variables to CAN (using subjects without CAN as a reference group). Potential confounding variables were controlled in the regression model. The predictive performance of the DM-HR was evaluated using the area under the curve (AUC) in a receiver-operating characteristics (ROC) curve. Odds ratios (ORs) with 95% confidence intervals (CIs) were calculated for the associations of DM, HR, or DM-HR with CAN. The results were analyzed using the Statistical Package for Social Sciences for Windows, version 16.0 (SPSS, Chicago, IL, USA). The tests were two sided, and a *P* value of <0.05 was considered significant.

## 3. Results

The baseline clinical characteristics of the 2,902 subjects were grouped according to CAN (Table 1). A total of 387 (18.51%) subjects had CAN. The prevalence of HT, DM, and MetS in the entire sample was 46.65%, 21.33%, and 39.82%, respectively. The entire sample included 705 men and 1,387 women (mean age 60.42 ± 8.68 years; Table 1). There was no significant difference in gender between subjects with CAN and those without CAN (male 32.96% versus 36.95%, *P* = 0.134). The HRV indices decreased with age (data not shown). The HR of subjects with CAN was very significantly higher than that of subjects without CAN (79.70 bpm versus 70.77 bpm, *P* < 0.001). Most HRV indices were lower in subjects with CAN compared with those without CAN (*P* < 0.01 for all).

### 3.1. CAN Prevalence

The CAN prevalence was 14.54% and 24.49% in subjects without DM and with DM, respectively. The CAN prevalence significantly increased in patients with the DM. CAN prevalence was 5.92%, 12.93%, 23.94%, and 53.67% in the respective groups according to HR. There was an increased CAN prevalence trend in groups with increased HR (*P* for trend < 0.001). In addition, CAN prevalence significantly differed among the groups according to the categorical variable of DM-HR (codes 1 for 5.33% and 7 for 66.67%). Interestingly, CAN prevalence was similar in subjects with DM-HR code of 2 and those with DM-HR code of 3 (9.09% versus 10.91%). Similar CAN prevalence was also found in subjects with DM-HR code of 4 and subjects with DM-HR code of 5 (21.25% versus 19.29%). As the DM-HR score increased, the CAN prevalence also increased.

### 3.2. Association Analysis for CAN

To estimate the potential risk factors of CAN, univariate analysis was performed in the entire sample. These potential risk factors contained the demographic parameters, blood glucose, and insulin function parameters as well as lipid profiles and medical history factors. The results indicate that these potential risk factors, including age, BMI, WC, SBP, DBP, HT, DM, MetS, FPG, PBG, HbAlc, FINS, IR, TG, and HR, were significantly associated with CAN (*P* < 0.05 for all parameters; Table 2). The two variables of HR and DM were very significantly associated with CAN in a univariate analysis model. To estimate the association of DM, HR, and DM-HR with CAN, MLR analysis was carried out to determine the extent to which CAN was associated with DM and HR. DM and HR remained very significantly associated with CAN after adjustments for age, gender, smoking, BMI, IR, TG, UA, and HT (*P* < 0.001 for DM and HR, resp.). In subjects with HR ranging from 65 to 75 bpm, the OR of CAN was 2.552 (95% CI, 2.216–2.938; Table 3) compared to subjects with HR less than 65 bpm. And in subjects with DM, the OR of CAN was 1.773 (95% CI, 1.337–2.351; Table 3) compared to subjects without DM. In addition, a very significant association between DM-HR and CAN was found by using MLR adjustment for potential confounds including age, gender, smoking, BMI, IR, TG, UA, and HT (*P* < 0.001). In subjects with DM-HR of 1, the OR of CAN was 1.607 (95% CI, 1.501–1.721; Table 3) compared to subjects with DM-HR of 0.

### 3.3. Predictive Value Analysis for CAN

**Figure 1:**
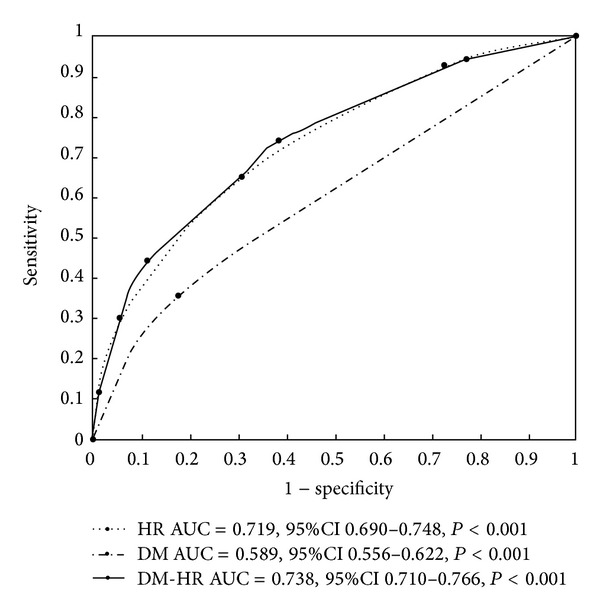
Receiver-operating characteristic curves showed the performance of resting heart rate (HR), diabetes (DM), and categorical variable of DM-HR in predicting cardiovascular autonomic neuropathy (CAN) prevalence in this dataset. The 95% confidence interval (CI) is given in parentheses. AUC represents area under the curve. HR: AUC = 0.719, 95% CI 0.690–0.748, *P* < 0.001; DM: AUC = 0.589, 95% CI 0.556–0.622, *P* < 0.001; DM-HR: AUC = 0.738, 95% CI 0.710–0.766, *P* < 0.001.

To evaluate the predictive performance of DM, HR, and DM-HR for CAN, the AUC in an ROC curve was calculated. For the DM variable, the AUC was 0.589 (95% CI: 0.556–0.622, *P* < 0.001), indicating that DM moderately predicted CAN, whereas for both the HR and DM-HR variables, the AUC was 0.719 (95% CI: 0.690–0.748, *P* < 0.001) and 0.738 (95% CI: 0.710–0.766, *P* < 0.001), respectively, suggesting that HR and DM-HR strongly predicted CAN (Figure 1). A cutoff point for MetS-HR was set to 4 of 7, and the sensitivity and specificity of CAN were 65.10% and 68.90% (Youden index = 0.340), respectively. The sensitivity and specificity of CAN were 73.90% and 61.50% (Youden index = 0.354), respectively, when the cutoff point was set to 3 of 7.

## 4. Discussion

A large-scale, population-based, cross-sectional study was conducted to evaluate the extent to which DM and resting HR are associated with CAN among 2,092 participants in the Chinese population. This sample was a good representation of the Chinese population, and the findings may work similarly well outside the areas studied in China. Importantly, in general Chinese population, we first performed predictive value analysis for CAN by using resting HR and DM. It is crucial for us to understand the predictive value of the two factors on CAN in the general population. This is partly because the DM prevalence has increased rapidly in China and may also contribute to CAN progression. Clinicians can expect to treat more DM patients having CAN progression. Moreover, a better understanding of the predictive value of the two factors will help clinicians in preventing and treating CAN.

The interesting finding was that resting HR alone or combined with DM both had a high value in predicting CAN in the general population. First of all, univariate and association analysis for CAN show that DM and resting HR are strongly and independently associated with CAN in the general population. Moreover, after adjustment for potential confounds, MLR analysis demonstrated that DM and resting HR very significantly and independently remain associated with CAN (*P* < 0.001 for both, Table 3). These results provided evidence that there is a good association of CAN with DM and resting HR.

Our previous study [13] and other previous studies [15–17] reported that DM and resting HR are significantly associated with CA function indices. Generally, increased resting HR was considered one of early indicators of CAN. Van Dijk et al. [18] conducted a study to explore the effects of baseline BP and heart frequency on autonomic function tests to show that HR was correlated with autonomic imbalance. Cardiovascular system is dually innervated, receiving fibers from the parasympathetic and sympathetic divisions of the autonomic nervous system. CAN manifests first in longer nerves. The vagus nerve, the longest of the autonomic nervous system, accounts for ~75% of all parasympathetic activity. The disease is ultimately the result of complex interactions among degree of glycemic control, disease duration, age-related neuronal attrition, and systolic and diastolic blood pressure [19]. The clustering of cardiovascular risk factors in DM indicates that the multiple complex metabolic reactions involved in glycotoxicity, lipotoxicity, altered insulin signaling, increased cytokine activity, and interstitial deposition of triacylglycerol may directly or indirectly affect CA function [15–17, 20]. Hyperglycemia plays the key role in the activation of venous biochemical pathways related to the metabolic and redox state of the cell, which contribute to the development and progression of CAN [21]. Basic medical studies implicated a number of pathogenic pathways that may impact autonomic neuronal function, such as formation of advanced glycation end products, increased free radical production, and activation of genes involved in neuronal damage [22, 23]. The exact mechanism underlying the association between CAN and DM or resting HR has not been fully elucidated. In the present study, we did not determine the mechanism by which DM modifies metabolic factors and induces CAN.

In addition, the predictive performance of DM and resting HR for CAN was evaluated by using AUC in receiver operative characteristic curve to show resting HR and DM-HR having high predictive value for CAN in general population. The AUC was calculated to show that resting HR strongly predicts CAN (AUC = 0.719, 95% CI: 0.690–0.748). For the analysis of the predictive value of DM alone on CAN, although association analysis showed that DM was very significantly and independently associated with CAN, the AUC was calculated to indicate that DM moderately predicts CAN (AUC = 0.589, 95% CI: 0.556–0.622). However, we used a categorical variable of DM-HR, which combined information between resting HR and DM, to signify a high value in predicting CAN in the general population (AUC = 0.738, 95% CI: 0.710–0.766). The sensitivity and specificity of CAN were 73.90% and 61.50% when the optimal cutoff point of DM-HR was set to 3 of 7. In particular, the CAN prevalence was 66.67% in subjects with resting HR > 85 bpm and DM (DM-HR = 8), while its prevalence decreased to 5.33% in subjects with resting HR < 65 bpm without DM (DM-HR = 1). These results supported that resting HR and DM-HR have a high value in predicting CAN in the general Chinese population. The DM-HR and resting HR cannot obtain a sensitivity of 100%. A false negative is mainly attributed to the fact that other risk factors contribute to the outcome. The natural history of CAN was sign of resting tachycardia due to impaired parasympathetic nervous systems in its early stage, and a normal resting HR would be detected due to both impaired sympathetic and parasympathetic nervous systems in the end of stage. In this study, some of the patients with CAN were not obese or had a normal resting HR due to the end of stage of CAN. Little is known about the CAN prevalence in subjects with a normal resting HR in the population. In addition, false-negative individuals had a lower resting HR, indicating that those people had long-term CAN. To our knowledge, this is the first study to have reported resting HR combined with DM having such a high predictive value for CAN in a Chinese population. This finding is of importance to the clinical practice of preventing and treating CAN in the general population.

Several limitations of this study deserve comment. First, the study design was cross-sectional, and thus the temporal sequence between risk factors and outcome was questionable. In addition, it is important to mention that our study was performed on Chinese individuals, and our findings may not be relevant to people of other ethnicities.

In conclusion, there was a tendency toward increased CAN prevalence with increased resting HR. Our findings signify that resting HR and DM are independently associated with CAN; and resting HR alone or combined with DM both have a high predictive value in predicting CAN in the general population. These observations provide novel insights into biological functions in the future.

## Figures and Tables

**Table 1:** Clinical characteristics of subjects.

Variable	Entire sample	Subjects without CAN	Subjects with CAN	*P* value*
Demographic information				
*N*	2096	1705	387	
Age (years)	60.42 ± 8.68	59.85 ± 8.64	62.94 ± 8.43	<0.001
Gender male, (%)	705 (33.7%)	562 (32.96%)	143 (36.95%)	0.134
BMI (kg/m^2^)	24.21 ± 3.37	24.07 ± 3.28	24.84 ± 3.7	<0.001
WC (cm)	85.07 ± 9.77	84.47 ± 9.62	87.72 ± 9.99	<0.001
SBP (mmHg)	127.62 ± 18.77	126.39 ± 18.22	133.05 ± 20.19	<0.001
DBP (mmHg)	79.83 ± 9.74	79.5 ± 9.65	81.31 ± 10.01	0.001
Medical history				
Smoking yes, (%)	306 (14.63%)	244 (14.31%)	62 (16.02%)	0.390
MetS yes, (%)	833 (39.82%)	629 (36.89%)	204 (52.71%)	<0.001
HT yes, (%)	976 (46.65%)	735 (43.11%)	241 (62.27%)	<0.001
DM yes, (%)	446 (21.33%)	307 (18.02%)	139 (35.92%)	<0.001
Laboratory measurement				
FPG (mmol/L)	5.53 ± 1.82	5.40 ± 1.58	6.12 ± 2.54	<0.001
PBG (mmol/L)	7.67 ± 3.63	7.36 ± 3.3	9.07 ± 4.6	<0.001
HbAlc (%)	6 ± 1.08	5.89 ± 0.92	6.47 ± 1.54	<0.001
FINS (*μ*ml/L)	7.19 ± 11.86	6.74 ± 8.03	9.18 ± 21.71	<0.001
IR (mmol/L)	1.81 ± 3.31	1.64 ± 2.13	2.54 ± 6.22	<0.001
TC (mmol/L)	5.32 ± 1	5.31 ± 0.98	5.39 ± 1.05	0.142
TG (mmol/L)	1.71 ± 0.98	1.67 ± 0.93	1.9 ± 1.17	<0.001
HDL (mmol/L)	1.36 ± 0.32	1.36 ± 0.33	1.34 ± 0.32	0.203
LDL (mmol/L)	3.19 ± 0.77	3.18 ± 0.76	3.23 ± 0.81	0.229
SCr (*μ*mol/L)	77.81 ± 26.11	77.65 ± 26.96	78.51 ± 21.98	0.561
UA (*μ*mol/L)	281.21 ± 84.01	280.13 ± 83.47	285.99 ± 86.26	0.216
HRV indices				
HR (bpm)	72.42 ± 10.13	70.77 ± 9.08	79.7 ± 11.26	<0.001
TP (ms^2^)	873.95 ± 702.47	1000.63 ± 693.2	315.87 ± 410.75	<0.001
LF (ms^2^)	190.98 ± 207.88	224.34 ± 215.08	43.97 ± 57.29	<0.001
HF (ms^2^)	183.05 ± 219.43	215.11 ± 229.61	41.82 ± 59.63	<0.001
LF/HF	1.70 ± 1.98	1.55 ± 1.48	2.37 ± 3.32	<0.001

Note: *presents the difference between subjects with and without cardiovascular autonomic neuropathy (CAN). BMI: body mass index; WC: waist circumference; SBP: systolic blood pressure; DBP: diastolic blood pressure; FPG: fasting plasma glucose; PBG: plasma blood glucose; FINS: fasting blood insulin; IR: insulin resistance; TC: serum total cholesterol; TG: triglyceride; UA: uric acid; HDL: high-density lipoprotein cholesterol; LDL: low density lipoprotein cholesterol; SCr: serum creatinine; HR: heart rate; TP: total power of variance; LF: low frequency; HF: high frequency; MetS: metabolic syndrome; DM: diabetes; HT: hypertension.

**Table 2:** Univariate logistic regression analysis for cardiovascular autonomic neuropathy.

Variable	*β*	SE	*P* value	OR	95% CI
Age	0.042	0.007	<0.001	1.04	1.029–1.103
Gender	0.176	0.117	0.134	1.19	0.947–1.547
BMI	0.066	0.016	<0.001	1.07	1.034–1.046
WC	0.034	0.006	<0.001	1.03	1.023–1.024
SBP	0.018	0.003	<0.001	1.02	1.012–1.030
DBP	0.019	0.006	0.001	1.02	1.007–1.261
Smoking	0.133	0.155	0.390	1.14	0.843–2.733
MetS	0.646	0.114	<0.001	1.91	1.527–1.307
HT	0.779	0.116	<0.001	2.18	1.736–3.248
HR	0.952	0.068	<0.001	2.59	2.267–2.565
DM	0.936	0.123	<0.001	2.55	2.003–3.156
DM-HR	0.487	0.033	<0.001	1.63	1.525–1.737
FPG	0.178	0.027	<0.001	1.19	1.133–1.149
PBG	0.111	0.014	<0.001	1.12	1.087–1.722
HbAlc	0.392	0.077	<0.001	1.48	1.271–1.026
FINS	0.014	0.006	0.015	1.01	1.003–1.159
IR	0.091	0.029	0.001	1.10	1.036–1.212
TC	0.082	0.056	0.142	1.09	0.973–1.369
TG	0.213	0.051	<0.001	1.24	1.119–1.129
HDL	−0.225	0.177	0.203	0.80	0.564–1.259
LDL	0.088	0.073	0.229	1.09	0.946–1.005
UA	0.001	0.001	0.216	1.00	0.999–1.108

Note: CAN: cardiovascular autonomic neuropathy; *β*: regression coefficient; SE: standard error; OR: odds ratio; CI: confidence interval; BMI: body mass index; WC: waist circumference; SBP: systolic blood pressure; DBP: diastolic blood pressure; FPG: fasting plasma glucose; PBG: plasma blood glucose; FINS: fasting blood insulin; IR: insulin resistance; TC: serum total cholesterol; TG: triglyceride; UA: uric acid; HDL: high-density lipoprotein cholesterol; LDL: low density lipoprotein cholesterol; SCr: serum creatinine; HR: heart rate; MetS: metabolic syndrome; HT: hypertension; DM: diabetes.

**Table 3:** Multivariate logistic regression analysis for cardiovascular autonomic neuropathy.

Model	Variable	*β*	SE	*P* value	OR	95% CI
Model 1	DM	0.573	0.144	<0.001	1.77	1.337–2.351
	HR	0.937	0.072	<0.001	2.55	2.216–2.938
Model 2	DM-HR	0.475	0.035	<0.001	1.61	1.501–1.721

Note: Model 1 and Model 2 adjusted for age, gender, smoking, BMI, IR, TG, UA, HT; *β*: regression coefficient; SE: standard error; OR: odds ratio; CI: confidence interval; HT: hypertension; HR: heart rate; BMI: body mass index; IR: insulin resistance; TG: triglyceride; UA: uric acid; DM: diabetes.

## Abbreviation

AUC: Area under curve
BMI: Body mass index
BSA: Body surface area
CI: Confidence intervals
CAN: Cardiovascular autonomic neuropathy
SCr: Serum creatinine
DBP: Diastolic blood pressure
DM: Diabetes
FPG: Fasting plasma glucose
HbAlc: Glycosylated hemoglobin
HDL: High-density lipoprotein cholesterol
HOMA-IR: Homeostasis model assessment insulin resistance estimate
HRV: Heart rate variability
HT: Hypertension
IDF: International Diabetes Federation
LDL: Low-density lipoprotein cholesterol
MetS: Metabolic syndrome
MLR: Multivariable logistic linear regression
OGTT: Oral glucose tolerance test
OR: Odds ratios
PBG: Postprandial blood glucose
ROC: Receiver operative curve
HT: Hypertension
BP: Blood pressure
TC: Serum total cholesterol
TG: Triglyceride
WC: Waist circumference
UA: Uric acid.
